# Multiple sclerosis: a narrative overview of current pharmacotherapies and emerging treatment prospects

**DOI:** 10.1007/s43440-024-00642-0

**Published:** 2024-08-23

**Authors:** Piotr Olejnik, Zuzanna Roszkowska, Sylwia Adamus, Kaja Kasarełło

**Affiliations:** 1 Chair and Department of Experimental and Clinical Physiology, Laboratory of Centre for Preclinical Research, Medical University of Warsaw, Warsaw, Poland; 2 Biomedical Physics Division, Faculty of Physics, University of Warsaw, Warsaw, Poland

**Keywords:** Multiple sclerosis, Disease-modifying therapy, Treatment targets, New therapies, Treatment strategies

## Abstract

Multiple sclerosis (MS) is a chronic autoimmune disease characterized by pathological processes of demyelination, subsequent axonal loss, and neurodegeneration within the central nervous system. Despite the availability of numerous disease-modifying therapies that effectively manage this condition, there is an emerging need to identify novel therapeutic targets, particularly for progressive forms of MS. Based on contemporary insights into disease pathophysiology, ongoing efforts are directed toward developing innovative treatment modalities. Primarily, monoclonal antibodies have been extensively investigated for their efficacy in influencing specific pathological pathways not yet targeted. Emerging approaches emphasizing cellular mechanisms, such as chimeric antigen receptor T cell therapy targeting immunological cells, are attracting increasing interest. The evolving understanding of microglia and the involvement of ferroptotic mechanisms in MS pathogenesis presents further avenues for targeted therapies. Moreover, innovative treatment strategies extend beyond conventional approaches to encompass interventions that target alterations in microbiota composition and dietary modifications. These adjunctive therapies hold promise as complementary methods for the holistic management of MS. This narrative review aims to summarize current therapies and outline potential treatment methods for individuals with MS.

## Introduction

Multiple sclerosis (MS) is a chronic autoimmune disorder caused by an inappropriate inflammatory response to myelin antigens, which leads to neurodegeneration [1]. Based on clinical course, MS may take relapsing-remitting (RRMS), primary progressive (PPMS), or secondary progressive (SPMS) forms, which constitute a continuum of pathophysiological processes leading to demyelination and axonal loss [2]. RRMS is associated with acute fluctuating neuroinflammatory processes, whereas progressive forms are related to chronic inflammation with gradual degeneration of the central nervous system (CNS), including both the brain and the spinal cord [2, 3]. MS affects mainly young adults between 20 and 40 years of age, with the advantage of approximately three women to one man [2, 4], and is the leading nontraumatic cause of disability in this age group, with a prevalence of approximately 36 per 100,000 people worldwide which continues to increase globally [5]. The first appearance of focal neurological symptoms, known as clinically isolated syndrome (CIS), should always be considered as a first flare-up of MS [2, 6]. However, the final diagnosis is based on the presence of lesions on magnetic resonance imaging (MRI) disseminated in space and time according to the McDonald criteria [7]. Because of multifocal damage to different areas of the white matter in the CNS, MS can cause various neurological symptoms. Amongst them most common are unilateral optic neuritis, sensation or movement disorders, and urinary incontinence [6]. In addition, up to 70% of people with active MS experience cognitive impairment [8].

Although many etiological hypotheses have been proposed, such as EBV infection, vitamin D insufficiency, smoking, obesity during adolescence, and genetic factors [9], the specific cause-and-effect relationship of MS is still not fully understood [10]. Nevertheless, the pathomechanism involves the migration of immunological cells across the blood-brain barrier (BBB) and an improper immune response and was first assigned to autoreactive T helper type 1 (Th1) lymphocytes. Further studies have shown that different T cell types, including Th17 and CD8^+^ T cells, also play an important role in the pathogenesis of MS [11]. Recently, the involvement of overreactive B cells [12] and microglia [13] in MS pathophysiology was described. Microglia seem particularly interesting in accordance with their role in progressive MS, as these cells drive and mediate the chronic inflammatory process, leading to subsequent axonal loss [3]. Additionally, the effects of the gut microbiota and its continued stimulation of the host immune system are currently under discussion [14].

Presently, MS is indisputably a manageable disease, and clinicians are commanding a vast number of disease-modifying therapies (DMTs), including oral and injection methods. Moreover, numerous symptom-controlling medications are available [15]. The invention of the first DMT was a milestone for neurologists, and it has changed the lives of countless people worldwide. Nonetheless, MS remains an incurable disease [16]. Although the cure for MS is closer than ever, it is still beyond the reach of the researchers. This narrative review article aims to provide a comprehensive summary of the currently available and emerging treatment methods that could enhance disease management and potentially contribute to the development of a cure for MS.

## Methods

To thoroughly analyze this topic, we searched pertinent literature via the PubMed database from its inception to May 10, 2024. Both experimental and clinical studies were included. The titles and abstracts were screened for key terms, such as ‘Multiple sclerosis’, ‘Experimental Allergic Encephalomyelitis’, ‘Treatment options’, ‘Clinical trials’, and ‘Treatment targets’. Furthermore, we manually reviewed references from relevant papers to ensure accurate coverage of the topic. Additionally, we analyzed ClinicalTrials.gov in search of currently recruiting clinical trials that have not yet been described. The literature search was independently performed by one of the three researchers.

### Currently available treatment methods

Medications dedicated to treatment of the MS include those for managing acute exacerbations, DMTs that alter the natural course of the disease (summarized in Table 1.), and symptomatic medicines that alleviate symptoms persisting after exacerbations (not described) [15].

**Table 1:** Currently available disease-modifying therapies for multiple sclerosis (MS)

Therapeutic agent	Approved indication	Administration route	Main mechanism of action	Clinical effectiveness	Clinical trial	Reference
Low- or moderate-effectiveness agents						
*Interferon β*	CIS, RRMS	Subcutaneous injection	Through JAK-STAT pathways	Reduced relapse rate to 33% and increased time to relapse to 5 months compared to placebo.	PRISMS trial	[25]
*Glatiramer acetate*	CIS, RRMS	Subcutaneous injection	Resemblance to MBP	Reduced relapse rate to 34–78% compared to placebo	GALA trial	[27, 30 ]
*Fumarates* (i.e., *dimethyl fumarate*)	RRMS	Oral	Multiway action caused by different metabolic pathways – Nrf2-related and Nrf2-idnepentent – increasing concentration of FoxP3^+^ T cells	Relative reduction to 53% in ARR	DEFINE trial	[32]
*Teriflunomide*	RRMS	Oral	Inhibiting the pyrimidine synthesis, by affecting mitochondrial dihydroorotate dehydrogenase	Relative reduction to 36% in ARR compared to placebo, significantly higher ARR compared to Interferon β	TOWER trial, TENERE trial	[34, 35]
**High-efficacy treatment agents**						
*S1P receptors modulators* (i.e., *fingolimod*, *siponimod*, *ozanimod*, *ponesimod*)	RRMS, SPMS	Oral	Metabolite resembling S1P, decreasing lymphocytes infiltration through BBB	Fingolimod: relative reduction of ARR to 48% compared to placebo. Siponimod: reduced risk of 3-month confirmed disability progression in patients with SPMS	FREEDOMS II trial	[38, 39 ]
*Cladribine*	RRMS	Oral	Nucleoside analog of deoxyadenosine	Relative reduction to 58% in ARR	CLARITY trial	[41]
*Mitoxantrone*	RRMS, SPMS	Intravenous injection	Inhibition of B and T lymphocytes proliferation by intercalation with DNA molecules	Relative reduction to 68% in ARR	MIMS trial	[45]
*Natalizumab*	RRMS, SPMS	Intravenous injection	Monoclonal humanized antibody anti-α_4_β_1_-integrin, inhibiting lymphocytes infiltration through BBB	Relative reduction to 68% in ARR	AFFIRM trial	[48]
*Alemtuzumab*	RRMS	Intravenous injection	Monoclonal humanized antibody anti-CD52, leading to antibody-dependent cell-mediated cytotoxicity	Reduced relapse risk to 59% compared to Interferon β	CARE-MS I trial	[52]
*Ocrelizumab*	PPMS, RRMS	Intravenous injection	Monoclonal humanized antibody anti-CD20, leading to antibody-dependent cell-mediated cytotoxicity	Reduced relapse risk to 47% compared to Interferon β (RRMS) and cumulative proportion of patients without confirmed disability progression of 73 at 96 weeks (PPMS)	OPERA II trial	[56]
*Rituximab*	RRMS (*off-label use*)	Intravenous injection	Monoclonal chimeric antibody anti-CD20, leading to complement-dependent cytotoxicity	80% relapse-free patients during 72-week, open-label, phase I trial, relative reduction to 19% in ARR compared to dimethyl fumarate	RIFUND-MS trial	[57, 59]
*Ublituximab*	RRMS	Intravenous injection	Monoclonal chimeric antibody anti-CD20, leading to antibody-dependent cell-mediated cytotoxicity	Relative reduction to 50–60% in ARR compared to teriflunomide	ULTIMATE I and II trials	[61]
*Ofatumumab*	RRMS	Subcutaneous injection	Monoclonal human antibody anti-CD20 leading to complement-dependent cytotoxicity	Relative reduction to 60% in ARR compared to teriflunomide	ASCLEPIOS II trial	[60]

*Abbreviations* ARR – annual relapse rate, BBB – blood-brain barrier, CD20 – cluster of differentiation 20, CD52 – cluster of differentiation 52, CIS – clinically isolated syndrome, DNA – deoxyribonucleic acid, MBP – myelin basic protein, Nrf2 – nuclear factor erythroid 2-related factor 2, PPMS – primary-progressive multiple sclerosis, RRMS – relapsing-remitting multiple sclerosis, SPMS – secondary-progressive multiple sclerosis

### Treatment of relapses

Managing disease flare-ups is based on the use of glucocorticoids, in the acute phase with intravenous methylprednisolone, and during less severe with oral drugs, such as prednisone at high doses [17, 18]. Additionally, intravenous immunoglobulin (IVIG) can also be administered during disease relapse [19]. In addition to pharmacological methods, therapeutic plasma exchange (TPE) is an approved approach for treating acute steroid-resistant relapses [20].

### Disease-modifying therapies

According to the European Medicines Agency (EMA), approximately 20 DMTs are currently registered, offering effective control of disease activity [21]. These therapies can be categorized based on their effectiveness, as measured by the Expanded Disability Status Scale (EDSS), annualized relapse rate (ARR), and MRI. Specifically, they are classified as low- or moderate-effectiveness treatment agents (LETAs) or high-efficacy treatment agents (HETAs). Furthermore, DMTs might be administered via different routes: orally (i.e., dimethyl fumarate), subcutaneously (i.e., interferon β), or intravenously (i.e., natalizumab). The choice of DMT should be guided by the effectiveness and safety profile of the specific molecule [22].

### Low- or moderate-effectiveness agents (LETAs)

Among LETAs, the interferon β (IFN-β), the first DMT ever registered for RRMS treatment, is still considered the first-line therapy for this type of MS [15, 23]. The mechanisms by which IFN-β causes immunomodulatory and antiproliferative effects are omnidirectional and not yet fully understood. IFN-β leads to the downregulation of major histocompatibility complex (MHC) II expression on antigen-presenting cells (APCs), mostly by modulation of JAK-STAT pathways. [24]. According to the PRISMS randomized, double-blinded, placebo-controlled clinical trial, treatment with IFN-β resulted in a reduction in the relapse rate by up to 33% and an extension of the time to the first relapse by up to 5 months, in comparison to placebo [25]. Similarly, another LETA medication, glatiramer acetate (GA), has immunomodulatory functions, mainly because it resembles myelin basic protein (MBP), and reduces the inflammatory response of T cells against actual myelin sheaths [26]. Moreover, studies have revealed that GA triggers the release of multiple neurotrophic factors, resulting in neuroregenerative effects [27]. Although the pilot study of GA demonstrated a significant decrease in exacerbations [28], with an estimated ARR reduction of approximately 78%, the subsequent clinical trials shown reduced efficacy [29]. For example, the GALA (phase 3, randomized, placebo-controlled parallel-group study) revealed a 34% reduction in relapse compared with the placebo [30]. Another LETA medications are fumarates, including dimethyl fumarate, which is primarily used to treat psoriasis and has a broad spectrum of anti-inflammatory activities. It modulates multiple metabolic pathways, like related to Nrf2 (nuclear factor erythroid 2-related factor 2), which has an antioxidant role, increases the concentration of FoxP3^+^ T cells and reduces the number of CD8^+^ T cells and B cells. Dimethyl fumarate may also act through Nrf2-independent pathways, thereby decreasing immune cell migration through the BBB [31]. The effectiveness of dimethyl fumarate, as demonstrated in the DEFINE randomized, double-blind, placebo-controlled phase 3 trial, resulted in a relative reduction of 53% in the ARR [32]. The physicians may also use another LETA, teriflunomide, an antiproliferative factor that inhibits pyrimidine synthesis by influencing mitochondrial dihydroorotate dehydrogenase. Teriflunomide affects rapidly dividing cells, including autoreactive lymphocytes, and by that regulates the inflammatory response in MS [33]. Although the TOWER randomized, double-blind, placebo-controlled phase 3 trial demonstrated a 36% reduction in the ARR compared with placebo [34], teriflunomide resulted in a significantly greater ARR than IFN-β [35].

### High-efficacy treatment agents (HETAs)

Another group of DMTs, HETAs comprises sphingosine-1-phosphate (S1P) receptor modulators, including fingolimod, siponimod (registered for SPMS treatment), ozanimod, and ponesimod [36]. Fingolimod was registered as the first treatment agent in this group. Its mechanism of action is based on imitating natural molecule, as fingolimod’s active metabolite, fingolimod-phosphate, is similar to S1P, which is crucial for the regulation of lymphocyte migration from the lymph nodes to the blood. Fingolimod leads to the downregulation of S1P receptors on lymphocytes, reducing their infiltration into the CNS, which subsequently exercise immunosuppression influence [37]. According to the FREEDOMS II double-blind, randomized, placebo-controlled, phase 3 trial, fingolimod caused a relative reduction in the ARR of up to 48% in comparison with placebo. Nonetheless, there was no statistically significant difference between the fingolimod and placebo groups in terms of confirmed disability progression, as measured by the EDSS [38]. The siponimod, which is approved for the treatment of both RRMS and SPMS, has been shown to reduce the risk of 3-month confirmed disability progression, also measured by the EDSS [39]. Furthermore, cladribine is a possible HETA for people with highly active RRMS. Cladribine was previously registered as a chemotherapy for numerous hematological neoplastic diseases and is used in autoimmune disorders such as celiac disease and MS. Cladribine, a nucleoside analog of deoxyadenosine, causes the depletion of autoreactive lymphocytes and, consequently, has an immunosuppressive effect [40]. The CLARITY phase 3, double-blind, placebo-controlled, multicenter trial reported an approximately 58% relative reduction in the ARR. However, this treatment is associated with statistically more frequent lymphocytopenia [41]. Not only cladribine but also mitoxantrone is an antineoplastic agent used for MS treatment. The main mechanism of action of mitoxantrone is through DNA intercalation eventuating in cross-links, which leads to cracks in the DNA strands [42], as it serves as a topoisomerase II inhibitor [43]. Mitoxantrone has long been considered an effective treatment for patients with aggressive RRMS and rapidly progressive SPMS [44]. According to the MIMS placebo-controlled, double-blind, randomized, multicenter trial, mitoxantrone was effective in decreasing the relative ARR by up to 68% [45]. Although Hartung et al. did not observe any clinically significant cardiac dysfunction, it is well established that mitoxantrone has cardiotoxic effects [46], therefore, it is rarely used. Fortunately, a group of monoclonal antibodies (mAbs) can also be administered for MS treatment. Among these HETAs, the first mAb ever registered for the treatment of MS was natalizumab. It is a humanized mAb that blocks α_4_β_1_-integrin, causing inhibition of the autoreactive lymphocyte’s migration through the BBB [47]. The AFFIRM phase 3, double-blind, placebo-controlled trial revealed a relative reduction in the relapse risk of up to 68%. Additionally, natalizumab decreased the risk of disability progression, as measured by the EDSS, compared with the placebo group [48]. Nevertheless, natalizumab is the medication most frequently associated with progressive multifocal leukoencephalopathy [49]. Another mAb used for the treatment of MS is alemtuzumab, which targets CD52. Little is still known about the CD52 antigen and its exact role in the immune response, however studies indicate that it is present on a vast spectrum of immune cells, including B and T lymphocytes, which plays major role in MS pathogenesis [50]. Thus, alemtuzumab leads to the depletion of these cell types and decreases disease activity [50, 51]. According to the CARE-MS I randomized, rater-masked, phase 3 trial, alemtuzumab reduced the relapse risk by up to 59% compared with IFN-β [52]. Finally, neurologists may use a wide variety of anti-CD20 agents, such as ocrelizumab, rituximab, ofatumumab, and ublituximab [53]. Their mechanism of action is based on their interaction with B lymphocytes and, consequently, B cells depletion. Anti-CD20 antibodies play a role in two different mechanisms. Complement-dependent cytotoxicity was observed for rituximab and ofatumumab, whereas antibody-dependent cell-mediated cytotoxicity was noted for ocrelizumab and ublituximab [54]. Although all these agents act similarly, ocrelizumab is worth highlighting, since it is the only medication registered for the treatment of PPMS up to date [55]. According to the OPERA II phase 3, multicenter, randomized, double-blind, active-controlled, parallel-group trial, ocrelizumab decreased the ARR by up to 47% compared with the IFN-β [56]. Additionally, a meta-analysis conducted by Sui et al. revealed that ocrelizumab had the highest efficacy in the treatment of PPMS, based on the ratio of patients without confirmed disability progression [57]. Although rituximab is widely used for the treatment of RRMS, it has not been registered to date. The 72-week, open-label, phase I trial conducted by Bar-Or et al. indicated that over 80% of patients remained relapse-free over the analyzed time period [58]. Additionally, the RIFUND-MS rater-blinded, phase 3, randomized controlled trial by Svenningsson et al. revealed that, compared with dimethyl fumarate, rituximab caused a relative reduction in the ARR of up to 19% [59]. Furthermore, another anti-CD20 mAb, ofatumumab, led to a relative reduction in the ARR of up to 60% compared with the teriflunomide, according to the ASCLEPIOS II double-blind, double-dummy, phase 3 trial [60]. The most recently registered DMT is ublituximab, which still lacks real-world clinical evidence. The primary findings concerning treatment efficacy were derived from the ULTIMATE I and II studies. According to those phase 3, double-blind, double-dummy trials, the relative reduction in ARR was approximately 60% and 50%, respectively, over teriflunomide [61].

### Potential treatment options

**Fig. 1:**
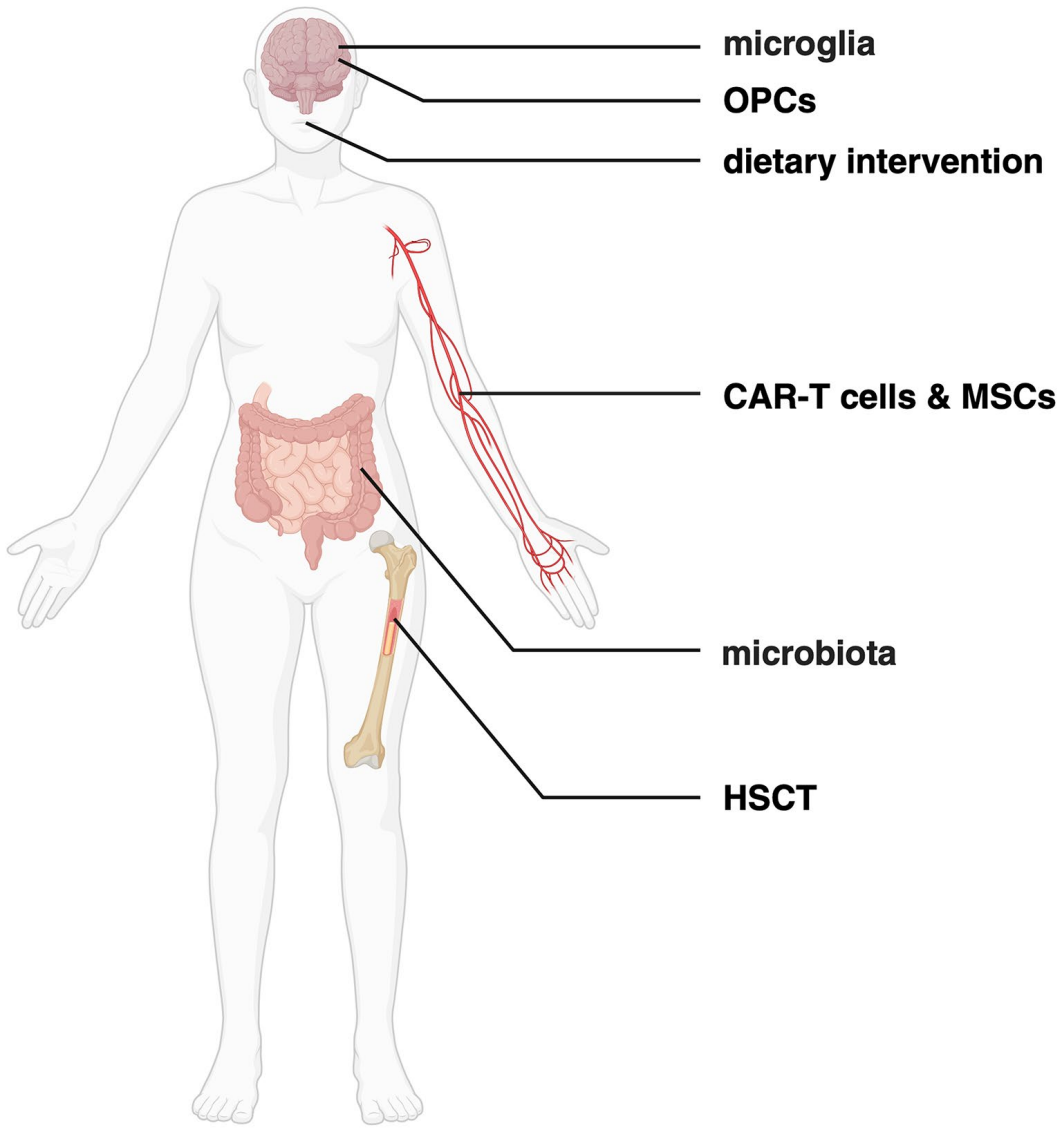
Potential therapeutic targets for novel treatments of MS. The treatment strategies involve depletion of microglia, the resident immune cells of the CNS, administration of OPCs, which show promise as the first remyelinating option in MS, or CAR-T cells targeting patients’ autoreactive immune cells. Moreover, HSCT may replace the overactive immune cells in patients, and MSCs are thought to play a neuroprotective and remyelinating role. Furthermore, interventions involving dietary modifications and alterations in microbiota composition are considered as complementary therapeutic strategies. The figure was created with BioRender.com. *Abbreviations CAR-T cells – chimeric antigen receptor T cells*,* CNS – central nervous system*,* HSCT – hematopoietic stem cell transplantation*,* MS – multiple sclerosis*,* MSCs – mesenchymal stem cells*,* OPCs – oligodendrocyte progenitor cells*

Despite the availability of various DMTs [15], a group of patients, particularly those with progressive forms remain unresponsive [23], and still are lacking effective treatment. This shows that current therapies offer only limited benefits [62]. This review highlights potential new treatments being under investigation (summarized in Fig. 1.), including approved but underutilized options like hematopoietic stem cell transplantation (HSCT), experimental approaches such as mAbs and chimeric antigen receptor T (CAR-T) cells, and still theoretical complementary methods like microbiota modification and dietary interventions. Table 2. summarizes both established therapeutic targets in clinical trials and those still in preclinical stages.

**Table 2:** Therapeutic approaches for multiple sclerosis (MS) evaluated in clinical and preclinical studies

Potential treatment options for multiple sclerosis			
Preclinically tested only	Reference	In clinical studies	Reference
OPCs therapy	[105]	monoclonal antibodies (i.e., Elezanumab, Temelimab, Inebilizumab)	[63, 75, 83]
		HSCT	[91–94]
ferroptosis	[108, 109]	MSCs therapy	[98–101]
		anti-CD19 CAR-T Cell Therapy	[116]
microglia depletion	[137, 138, 139]	BTK inhibitor	[134]
		FMT	[153, 154, 155]

*Abbreviations* BTK – Bruton’s tyrosine kinase, CAR-T Cell – chimeric antigen receptor T cell, CD19 – cluster of differentiation 19, FMT – fecal microbiota transplant, HSCT – hematopoietic stem cell transplantation, MSCs – mesenchymal stem cells, OPCs – oligodendrocyte progenitor cells

### Monoclonal antibodies

#### Elezanumab

Elezanumab (ABT-555) is a human antibody directed against repulsive guidance molecule A (RGMa) [63]. This molecule is expressed in the CNS and other tissues in adult organisms, but its high expression in mouse embryos indicates that RGMa plays a significant role in CNS development [64]. RGMa interferes with regenerative processes after CNS inflammatory or traumatic injury by inhibiting the outgrowth of axons and restoration of oligodendrocytes [63, 65]. Research has demonstrated that RGMa is involved in regulation of the integrity of BBB, as observed in animals with induced experimental autoimmune encephalomyelitis (EAE, an animal model of MS) and in human brain microvascular endothelial cell cultures. RGMa levels increase during EAE progression, and at the same time, the levels of zonula occludens 1 (ZO-1) and claudin-5, both tight junction components, decrease. In individuals with acute MS, serum RGMa levels are significantly elevated compared to healthy controls [66]. Postmortem analysis of tissues isolated from patients with progressive MS has shown the presence of RGMa in active chronic lesions [67].

Recent research has identified RGMa as a potential target for novel MS drugs. Studies conducted in animals with evoked EAE has shown that treatment with an antibody directed against RGMa decreased the disease severity and mortality of affected mice [65]. In another study, neutralization of RGMa by an anti-RGMa antibody resulted in a partial reduction in disease progression in NOD EAE mice model with secondary progressive MS. Moreover, anti-RGMa antibodies have been shown to mitigate axonal degeneration and demyelination in the spinal cords of EAE animals [68].

The single ascending dose (SAD) phase 1 study and the multiple ascending dose (MAD) phase 1b study (ClinicalTrials.gov Identifier: NCT02601885, all described clinical trials are summarized in Table 3.) were conducted to investigate the potential of elezanumab as a treatment option for relapsing MS. A decrease in free RGMa levels in the CSF of patients treated with the MAD protocol compared with placebo was reported on day 113, with elevated doses of elezanumab further decreasing free RGMa levels. Nonetheless, MRI assessment revealed no significant effect on the formation of new brain lesions. Elezanumab was found to be generally safe, well-tolerated, and did not raise significant concerns regarding its immunogenicity [63].

**Table 3:** Ongoing clinical trials investigating new therapies for multiple sclerosis (MS)

Agent	Mechanism of action	Indication	Clinical trial identifier*
ABT-555 (Elezanumab)	Monoclonal antibody against RGMa	RRMS, SPMS	NCT02601885 Phase I study
		PPMS, SPMS	NCT03737812 Phase II study
		RRMS, SPMS	NCT03737851 Phase II study
GNbAC1 (Temelimab)	Monoclonal antibody against HERV-W-ENV	RRMS, SPMS	NCT05049161 Phase II study
		RRMS, SPMS	NCT03239860 Phase II study
		RRMS	NCT02782858 Phase II study
		PPMS, RRMS, SPMS	NCT01639300 Phase II study
		RRMS, SPMS	NCT04480307 Phase II study
MEDI-551 (Inebilizumab)	Monoclonal antibody against CD19 molecule	CIS, RRMS, SPMS	NCT01585766 Phase I study
MSCs therapy	Cell-based therapy	PPMS, RRMS, SPMS	NCT02239393 Phase II study
Anti-CD19 CAR-T Cell Therapy	CAR-T Cell Therapy	All MS types	NCT06138132 Phase I study
SAR442168 (Tolebrutinib)	BTK inhibitor	PPMS, RRMS, SPMS	NCT06372145 Phase III study
		RRMS, SPMS	NCT03889639 Phase II study
FMT	Eubiosis restitution therapy	RRMS, SPMS	NCT03183869 Phase II study

*Abbreviations* BTK – Bruton’s tyrosine kinase, CAR-T Cell – chimeric antigen receptor T cell, CD19 – cluster of differentiation 19, CIS – clinically isolated syndrome, FMT – fecal microbiota transplantation, HERV-W-ENV – envelope protein of the human endogenous retrovirus type W, MSCs – mesenchymal stem cells, MS – multiple sclerosis, PPMS – primary-progressive multiple sclerosis,, RRMS – relapsing-remitting multiple sclerosis, RGMa – repulsive guidance molecule A, SPMS – secondary-progressive multiple sclerosis

* **ClinicalTrials.gov**

Additionally, two phase 2 clinical trials have been completed, investigating elezanumab as a treatment option for patients with progressive MS (ClinicalTrials.gov Identifier: NCT03737812) and as an adjunct therapy for relapsing MS in accordance with standard treatment guidelines (ClinicalTrials.gov Identifier: NCT03737851), but their results are pending publication.

#### Temelimab

Temelimab (GNbAC1), a humanized mAb class IgG4, targets the envelope protein of the human endogenous retrovirus type W HERV-W-ENV [69]. Human endogenous retroviruses (HERVs) are integrated into the human genome, and their RNA encodes proteins involved in the pathogenesis of diseases such as amyotrophic lateral sclerosis (ALS) and MS. Its expression can be triggered by various factors, e.g., viruses such as the Epstein–Barr virus or proinflammatory cytokines [70].

One of the HERV proteins is the envelope protein, which is abnormally expressed in the active brain lesions of people with MS, with positive immunolabeling observed in activated macrophages, microglia, and, to some extent, astrocytes [71]. HERV-W-ENV can modulate microglial function in several ways, e.g., by promoting differentiation into the M1 proinflammatory phenotype and decreasing the expression of neuroprotective factors [72].

In mice with EAE, GNbAC1 has been shown to inhibit the release of proinflammatory cytokines (IL-6 and TNF-α) stimulated by the envelope protein [73]. Additionally, GNbAC1 administration to oligodendrocyte precursor cell cultures stimulated by the envelope protein reduced the transcription of inducible nitric oxide synthase (iNOS), thereby decreasing nitrosative stress [74].

To date, six clinical trials considering temelimab as a treatment for MS have been conducted. Two phase 2 studies were ultimately terminated for independent reasons (ClinicalTrials.gov Identifier: NCT05049161, ClinicalTrials.gov Identifier: NCT03239860). Completed studies include a phase 2 safety study in patients with PPMS, SPMS, and RRMS (ClinicalTrials.gov Identifier: NCT01639300) and the CHANGE-MS study (ClinicalTrials.gov Identifier: NCT02782858). CHANGE-MS followed by the prematurely terminated extension study ANGEL-MS (ClinicalTrials.gov Identifier: NCT03239860) included patients with active RRMS, with approximately 90% of the subjects not receiving DMTs before enrollment. No significant differences were found in the number of MRI-assessed gadolinium-enhanced T1-weighted lesions between temelimab and placebo groups after 24 weeks. Nevertheless, temelimab demonstrated neuroprotective features, by preventing brain volume loss, stabilizing the magnetization transfer ratio signal as indicative of myelin integrity, and reducing the number and size of newly formed T1-hypointense lesions at 48 and 96 weeks [75]. Also, additional clinical trial evaluating temelimab as a potential MS treatment has been undertaken in the last few years, involving its administration following rituximab therapy and extending the range of temelimab doses (ClinicalTrials.gov Identifier: NCT04480307), though its results are yet to be published.

#### Inebilizumab

As the involvement of B cells in the pathomechanism of MS is well known [12, 76], targeting B cells has garnered significant interest, leading to the approval of mAbs directed against CD20 for MS treatment [53, 77]. In recent years, CD19 has emerged as another promising target in the treatment of relapsing MS [78]. The expression of CD19 begins in pre-B lymphocytes and is present in mature B cells, including plasma cells [79]. It has been reported that the total population of CD19^+^ B cells is elevated in people with MS compared to healthy controls, providing a rationale for the development of anti-CD19 therapy [80].

Inebilizumab (MEDI-551) is a humanized, afucosylated IgG1 mAb directed against CD19, resulting in CD19^+^ B cell depletion [81]. Research conducted in B-cell-dependent EAE mice induced by immunization with recombinant human myelin oligodendrocyte glycoprotein (rhMOG), revealed that the administration of ineblizumab was associated with decreased severity of disease and lower leukocytes infiltration into the spinal cord [78]. Another study involving B-cell-dependent EAE mice model induced with humanized MOG, revealed that treatment with an anti-CD19 antibody diminished disease severity and was more effective than treatment with an anti-CD20 antibody. Moreover, anti-CD19 mAb administration led to a more significant reduction in anti-MOG IgGs in both serum and spinal cord compared to anti-CD20 mAb treatment [82].

To date, only one clinical trial investigated the use of a mAb against CD19 in people with relapsing MS (ClinicalTrials.gov Identifier: NCT01585766). In addition to the satisfactory safety and tolerability of inebilizumab, treated patients exhibited fewer new brain lesions compared to placebo group, with complete and rapid B cell depletion observed across all tested doses. Higher doses of the mAb were associated with more prolonged B cells depletion in patients. Additionally, a decrease in the number of circulating plasma cells was noted after treatment with inebilizumab [83].

### Cell-based therapies

#### Hematopoietic stem cell transplantation

HSCT has been used to treat autoimmune diseases since 1995 [84]. This procedure aims to destroy the immune system of the patient with ablative conditioning and to reconstitute it via hematopoietic stem cell administration [85]. In autologous HSCT (AHSCT), the patient’s cells are used [86].

According to the European Society for Blood and Marrow Transplantation guidelines published in 2020, AHSCT is currently established as a standard of medical care for patients up to 45 years old presenting highly active (evidenced by new relapses or new lesions within 12 months) RRMS that lasts no more than 10 years and does not respond to standard DMTs. Furthermore, for other types of MS, including progressive MS, aggressive MS and pediatric MS, the AHSCT should be considered as a clinical option, although in the child population it is important to first consider standard DMT, such as interferon or fingolimod, which present less severe toxicity [87].

The results of studies performed in MS patients are generally consistent and indicate that following AHSCT, there is a reduction in the number of autoreactive effector T cells, predominantly Th17 and CD8^+^, and a transient increase in the Treg population in the peripheral blood [85, 88, 89]. Many studies have confirmed the efficacy of AHSCT, both for RRMS and SPMS treatment. It seems reasonable that HSCT may not only decrease inflammatory activity but also delay the neurodegenerative process in SPMS, hence, neurofilament light chain levels decline after administration [90]. According to the phase I/II study conducted by Burt et al., clinical improvement in neurological disability, after the HSCT transplantation, was observed in RRMS patients who did not respond to IFN-β therapy [91]. Compared with standard DMTs, the MIST randomized clinical trial, which was conducted on 110 patients with RRMS, revealed that nonmyeloablative HSCT extended the time to disease progression. Additionally, the mean EDSS score declined after HSCT in comparison to the DMT group [92]. In 2022, Mariottini et al. confronted the efficacy of AHSCT in patients with SPMS to that of low-dose immunosuppression with intravenous cyclophosphamide (Cy). While patients receiving AHSCT exhibited a reduction in relapses, no discernible benefits of AHSCT over Cy in terms of disability severity was observed [93]. In 2023, Boffa et al. reported that the use of ASHCT in people with active SPMS slows the progression of disabilities and increases the likelihood of disability improvement in patients compared with standard DMTs [94].

#### Mesenchymal stem cell therapy

Mesenchymal stem cells (MSCs) are multipotential cells present in nearly all tissues capable of differentiating into various cell types. In recent years, there has been considerable interest in utilizing MSCs for regenerative therapy [95]. The MSCs might play a role in MS treatment in different ways. According to a study by Zhang et al., MSCs exhibit neuroprotective effect in EAE mice, as the administration of MSCs led to elevated levels of nerve growth factors and improved functional outcomes [96]. Other studies, including that conducted by Akiyama et al., suggest that MSCs might play a role in remyelination, but the precise mechanism remains unclear [97]. Despite these promising results, clinical trials delivered mixed results. The phase II randomized controlled trial by Tremblay et al., reported people with MS experienced no significant improvement following intravenous MSC therapy (ClinicalTrials.gov Identifier: NCT02239393) [98]. Likewise, randomized controlled trials by Nabavi et al. and Fernandez et al. did not reveal any improvement in patients’ neurological status after MSCs delivery [99, 100]. Similarly, the MESEMS phase 2, randomized, double-blind crossover trial conducted by Uccelli et al. on a vast group of 144 patients, emphasized that MSCs are not effective in the treatment of active MS [101]. Conversely, the latest meta-analysis revealed that more than 40% of people with MS treated with MSCs presented clinical improvement. However, the majority of the studies included in this analysis were not randomized and had negligible populations. Additionally, the sources of MSCs varied among studies, hence they cannot be compared directly [102].

#### Oligodendrocyte progenitor cell therapy

There are numerous treatment methods and targets, although most of them focusing on immunosuppression and decreasing disease activity. To date, there is no registered treatment particularly concentrating on the induction of remyelination, however there is potential in oligodendrocyte progenitor cells (OPCs) therapy [103]. Oligodendrocytes are the main CNS glial cells that are responsible for myelin sheath formation. Given that myelin sheaths are destroyed in the pathophysiology of MS, OPCs therapy is considered promising for their restoration [104]. Nevertheless, so far, there are many obstacles, such as the source of OPCs, route of administration (direct or intrathecal injection), infiltration into the CNS (as they generally do not cross the BBB), cell migration to affected areas, dosing, and the need for continuous immunosuppression [95]. According to a study by Mikaeili Agah et al., the course of EAE was milder in mice after OPCs transplantation compared to control mice [105]. In contrast, a study conducted by Chang et al. undermined the usefulness of OPCs by showing the presence of oligodendrocytes in chronic MS lesions without features of remyelination. Therefore, remyelination is influenced not only by the availability of oligodendrocyte progenitor cells and their capacity to differentiate into oligodendrocytes but also by additional factors that may affect the regeneration of the myelin sheath [106]. Thus, further investigations are warranted to evaluate the efficacy of OPCs in the treatment of MS.

#### Ferroptosis

Recent studies revealed that ferroptosis, which is an iron-mediated form of programmed cell death, might be involved in neurodegeneration and disease exacerbations in progressive MS [107]. The experiment conducted by Luoqian et al., through analysis of iron levels and the expression of ferroptosis-related proteins, highlighted the activation of ferroptotic pathways in the spinal cords of mice with EAE [108]. Another study by van San et al., confirmed these findings. Moreover, scientists revealed that the inhibition of ferroptosis (by UAMC-3203, highly soluble ferrostatin analog) significantly decreased the progression of EAE in mice [109]. Due to the lack of therapies capable of mitigating degenerative mechanisms in progressive forms of MS, the exploration of ferroptosis inhibitors has emerged as a compelling direction for investigation [110].

#### CAR-T cells

CAR-T cells are genetically engineered human T lymphocytes expressing receptors programmed to specifically recognize particular antigens on the patient’s cells, subsequently leading to cytotoxicity. In the last few years, there has been considerable interest in CAR-T cell treatment for malignancies, which has resulted in the registration of several therapies [111]. Thus, whether CAR-T cell therapy could be useful in treating autoimmune disorders [112], including MS [113], is still uncertain. Although mAbs and CAR-T cells play similar roles, CAR-T cells might outperform antibodies, as they are longer lasting and able to self-divisions [114].

Undoubtedly, the largest watershed in MS treatment was the invention of anti-CD20 agents, which are capable of B cell depletion. Accordingly, studies on CAR-T cells that target B cells in MS models exist and are very promising. In one of the latest studies conducted by Gupta et al., anti-CD19 CAR-T cells caused depletion of B cells in EAE mice [115]. There have been two cases reported by Fischbach et al., where they observed that anti-CD19 CAR-T cells were safe and were able to decrease the IgG concentration in patients’ CSF [116]. Furthermore, there is one registered clinical trial designed at Stanford University to assess the efficacy of anti-CD19 CAR-T cells in the treatment of progressive forms of MS (ClinicalTrials.gov Identifier: NCT06138132). The study has not yet recruited patients.

In addition to B cells, different targets of CAR-T cells have been studied as potential treatments for people with MS. Moorman et al. created CAR-T cells and CAR-Tregs aimed at type one dendritic cells (DCs) and assessed their impact on mice with EAE [117]. The rationale for this study was to target DCs that serve as antigen-presenting cells, what subsequently causes the indirect inactivation of autoreactive T cells [118]. Unfortunately, according to Moorman’s study, the effectiveness of DC-targeted CAR-T cells mishappened in depletion of DCs in immunocompetent mice [117].

Prospectively, CAR-T cells might play a greater role in MS treatment, especially in progressive MS types, for which treatment options are still lacking. However, further research is required to assess their safety and effectiveness.

#### Microglia

Microglia are a specialized subpopulation of phagocytes and APCs residing in the CNS and originating from the yolk sac during embryogenesis. These cells are essential for maintaining homeostasis in the developing and adult CNS. During development, microglia are believed to support neurogenesis and regulate axonal growth. In the adult brain, microglia maintain regulatory and developmental functions by participating in synaptic plasticity and myelination by influencing oligodendrocytes and their progenitors. [119, 120]. Thus, microglia are considered a key therapeutic target in diseases that disrupt myelin, such as MS [121].

Microglia present a broad spectrum of phenotypes between M1, the proinflammatory subtype, and M2, described as anti-inflammatory and neuroprotective [120, 122]. Remarkably, research has revealed that in a healthy human brain, microglia exhibit an activation state within the white matter, which becomes more pronounced with age. This increase in microglial activation may be a natural aspect of aging. In MS patients, microglial activation was significantly higher in normal-appearing white matter compared to controls and further escalated as the disease progressed [123]. Analysis of postmortem brain samples from MS patients revealed that multiple microglia-like cells, mostly in the proinflammatory state of activation, are present in active lesions. Inactive lesions, without inflammation, contain few microglia cells, which begin to re-express P2RY12, a homeostatic marker not found in activated microglia, suggesting that their reappearance is connected to lesion inactivity [123]. Additionally, in autopsy samples from patients with progressive MS the preference for the polarization of microglia toward the M1 phenotype in slowly expanding lesions was found, suggesting the importance of M1 microglia in lesion development and progression [124].

Recent studies have shown that residential CNS cells are predominantly engaged in immune mechanisms underlying progressive MS [125]. Currently, the available treatment strategies for RRMS and SPMS are based on immunosuppressive drugs, which have limited efficacy in treating progressive MS [3, 125]. Hence, microglia could become important targets in the treatment of progressive MS.

#### Bruton’s tyrosine kinase inhibitors

Burton’s tyrosine kinase (BTK) is a TEC kinase involved in immune function regulation, particularly in B cell differentiation and signaling [126]. In addition to being expressed in B cells, it is expressed in other types of immune cells, such as T cells, mast cells, dendritic cells, macrophages and microglia. In microglia, BTK modulates phagocytosis, which may influence myelin damage in MS [126, 127].

Studies have shown that BTK is expressed in various CNS resident cells, including oligodendrocyte precursor cells, microglia, astrocytes, neurons, and immature oligodendrocytes, with significantly higher expression levels in microglia. BTK expression was increased in mice in which central demyelination was induced by cuprizone. Additionally, analysis of postmortem brain tissue samples obtained from patients with progressive MS revealed that active and chronic active white matter lesions are characterized by increased BTK expression compared with inactive and remyelinating lesions [128].

Bruton tyrosine kinase inhibitors (BTKIs) are small molecules with the potential to cross the BBB, which is a limitation for many existing DMTs. This advantage allows the straightforward effect on the activity of immune cells in the CNS compartment [129]. In mice with secondary progressive EAE, inhibition of BTK with ibrutinib resulted in a decrease in disease severity, diminished demyelination, and reduced axonal degeneration. The administration of ibrutinib in the late stage of the disease decreased the number of myeloid cells infiltrating into the spinal cord and influenced the state of microglia by reducing the expression of several proinflammatory markers and increasing the expression of anti-inflammatory markers [130]. A subsequent investigation confirmed that different BTK inhibitors, such as remibrutinib, can reduce the severity of EAE in both humanMOG- and ratMOG-induced disease in mice. In rat MOG-induced EAE, BTK inhibition directly decreases the proinflammatory features of microglia [131].

Several clinical trials have been designed to assess the potential of BTKIs as MS therapeutics. Five inhibitors differing in selectivity, mode of binding, occupancy of the target and penetration into the CNS were included in a total of 14 ongoing clinical trials [132] (ClinicalTrials.gov Identifier: NCT06372145 for tocilibrutinib). Promising features of BTK inhibition in MS, such as the ability to decrease the number of gadolinium-enhanced lesions and T2 lesions, and to reduce the number of annual relapses, have already been observed for tolebrutinib (ClinicalTrials.gov Identifier: NCT03889639) [132, 133].

#### Microglia depletion

Since microglia cells are engaged in MS pathology, their depletion has become a target of interest as a potential treatment option for MS. Microglia depletion is mostly based on the inhibition of the class III tyrosine kinase receptor (colony-stimulating factor 1 receptor, CSF-1R) expressed by all types of microglia [134, 135]. Pharmacological microglial depletion via the inhibition of CSF-1R in EAE mice resulted in a reduction in disease severity, diminished demyelination in the spinal cord and indirectly increase in the number of mature oligodendrocytes [136]. In mice with mutations in the oligodendrocyte PLP1 gene, which mimics progressive MS neuroinflammation, CSF-1R inhibition reduces demyelination, neuronal and axonal degeneration, and indirectly affects T cell infiltration into the CNS [137].

However, several detrimental effects of pharmacological microglial depletion have also been reported. For example, in a mouse model of SPMS, microglial depletion increased disease progression and increased mortality. This strategy also induced inflammation, spurred CD4^+^ T cell proliferation, and infiltration into the CNS, what resulted in axonal damage and exacerbation of demyelination [138]. Thus, while microglial depletion holds potential as a MS treatment, it requires further investigation to address these risks [134]. To date, there have been no clinical trials assessing pharmacological microglial depletion in MS, but this strategy has been evaluated in other neurodegenerative diseases, such as Alzheimer’s disease (ClinicalTrials.gov Identifier: NCT04121208) and ALS (ClinicalTrials.gov Identifier: NCT04066244).

#### Gut microbiota

The gut microbiota encompasses a diverse range of microorganisms, including bacteria, archaea, fungi, eukaryotes, and viruses, that inhabit the human intestine [139, 140]. These commensal organisms can enhance both proinflammatory and regulatory responses in the body. Recent studies have shown that changes in the gut microbiome are an environmental risk factor for the development and progression of various diseases, including MS [139, 141]. The dominant phyla of the human microbiota are Firmicutes and Bacteroides. When a patient has a high Firmicutes/Bacteroides ratio, a state of dysbiosis occurs, which can lead to many inflammatory or metabolic disorders [142]. Cosorich et al. compared the microbiota of healthy volunteers with those of patients presented with RRMS. The results revealed decreased *Prevotella* strain levels in people with RRMS with highly active disease. It has been linked to increased levels of Th17 cells, which contribute to the development of inflammation [143]. Importantly, the microbiota metabolites of the *Prevotella* strain are known to produce propionate, a metabolite with anti-inflammatory properties [143, 144]. In a study conducted by Mikyake et al., the number of bacteria belonging to the *Clostridium* genus was decreased in patients with MS [145]. *Clostridia* strains were shown to increase the number of IL10-producing Tregs in mice [145, 146]. In general, many changes in the microbiota composition are present in MS patients. At the phylum level, the Euryarchaeota and Verrucomicrobia levels are increased in MS patients. On the other hand, decreases in *Butyricimonas* and *Prevotella*, genera from the Bacteroides phylum, and *Collinsella* and *Slackia*, genera from the Actinobacteria phylum, were observed in the study of Jangi et al. [147].

The gut microbiota affects the functioning of the intestines at many levels, including the regulation of gut permeability. Dysbiosis may be the cause of the uncontrolled passage of bacteria and bacterial products across the intestinal epithelium, which can trigger autoimmune responses that exacerbate MS [148].

Collectively, these studies highlight the importance of proper microbiota composition in the human intestine. Moreover, these findings suggest a new possible approach for MS treatment [149].

#### Fecal microbiota transplantation

The aim of fecal microbiota transplantation (FMT) is to restore the physiological microbiota by transferring stool from a healthy donor to the recipient. FMT is currently used to treat recurrent *Clostridioides difficile* infections and has garnered interest for its potential therapeutic utility in several other disorders [150, 151].

Al et al. reported that FMT can decrease small intestine permeability and enrich the gut microbiota in MS patients [152]. Additionally, a case report authored by Makkawi et al. described a 61-year-old woman with SPMS who presented with a gradually increasing EDSS score and recurrent *Clostridioides difficile* infections. Due to several unsuccessful attempts to treat these infections, it was decided to conduct FMT. Her EDDS score stabilized as soon as the procedure was done and slightly improved over the following 10 years [153].

Although single cases have revealed the beneficial effects of FMT on gastrointestinal function, preclinical investigations exploring the underlying mechanisms linking autoimmune responses to FMT in MS remain nascent. There have been several clinical trials, including one randomized, open-label, phase II study (ClinicalTrials.gov Identifier: NCT03183869, terminated due to nonmedical conditions, the results of which were inconclusive) [152], no consensus regarding the administration of FMT in MS treatment was achieved [154].

#### Diet interventions

Overall, the role of diet and nutritional supplements should be acknowledged as integral components of MS treatment. It can enhance the therapeutic efficacy of MS treatments, not only due to the direct influence of diet components on MS course, but also as an approach that impacts the composition of the gut microbiota. Munger et al. reported that the risk of MS is particularly low in white individuals with increasing serum levels of 25-hydroxyvitamin D, which is an inactive form of vitamin D. Vitamin D is a powerful immunomodulatory molecule that plays a role in several immune processes, both the innate and adaptive. Notably, the examined obese individuals had decreased levels of this metabolite [140, 155].

Interestingly, saturated fats impact the immune system through the activation of proinflammatory toll-like receptors. Toll-like receptor 4 also enhances the transport of bacteria-derived endotoxins in the intestines. Increased levels of such endotoxins may induce systemic inflammation in organisms, which may contribute to the development of metabolic or inflammatory diseases, such as autoimmune diseases [156, 157].

Wu et al. suggested that a high intake of salt can modulate serum glucocorticoid kinase 1, resulting in increased differentiation of proinflammatory Th17 cells, which are known to be involved in autoimmune processes. Moreover, mice fed with a high-salt diet developed more severe EAE than control mice [158]. Similar results were obtained by Kleinewietfeld et al., who reported that feeding animals with a high-salt diet increases the severity of EAE in mice and the number of Th17 cells in animals. Naïve human CD4^+^ T cells cultured under high-salt conditions presented increased production of IL-17 A, what resulted in the differentiation of Th17 cells, in which the upregulation of GM-CSF, TNFα and IL-2 was reported [159]. Nonetheless, to date, there is still a lack of unambiguous evidence on the effects of diet on the MS course, and large prospective clinical trials are needed [160].

#### Impact of diet on the gut microbiota

It is conceivable that the composition of the microbiota can be modified through dietary interventions since nutrients have beneficial effects on the microbiome. Fiber, omega-3 unsaturated fatty acids, or vitamin D3 promote the growth of microorganisms that generate anti-inflammatory factors [140]. In contrast, a diet rich in fats, refined sugar, and low fiber content have the potential to induce a chronic state of inflammation in the gastrointestinal system [140, 149]. Obesity is a well-known risk factor for MS [9]. Turnbaugh et al. investigated lean and obese twins and revealed an association between obesity and unfavorable changes in the microbiome, including diminished bacterial diversity. Additionally, a study revealed that obese patients have reduced levels of Bacteroidetes, what also occurs in people with MS [147, 161].

#### Conclusions and future perspectives

Overall, there are many potential treatment options for MS but, still MS remains an incurable disease. Importantly, there are still only few medications for progressive forms of MS, and this problem should undoubtedly be addressed. Although, an increasing number of therapeutic targets, including microglia, the microbiota, numerous cell-based therapies (HSCT, OPC, MSC, and CAR-T cells), ferroptotic mechanisms, and even diet interventions, are being studied, the clinical evidence for most of them are still limited. Definitely, there is a need for large, randomized clinical trials, and finally the real-world data describing effectiveness of researched therapeutics.

## Study limitations

The article addresses a broad and complex topic that necessitates certain simplifications due to the form of the article. Consequently, the most significant limitation of our study is that some aspects of the analysis may be overly superficial, and we could have inadvertently omitted some potentially interesting and promising treatment methods.

## Data Availability

No datasets were generated or analysed during the current study.

## Abbreviations

AHSCT: Autologous hematopoietic stem cell transplantation
ALS: Amyotrophic lateral sclerosis
APC: Antigen-presenting cell
ARR: Annual relapse rate
BBB: Blood-brain barrier
BTK: Bruton’s tyrosine kinase
BTKI: Bruton’s tyrosine kinase inhibitor
CAR-T cells: Chimeric antigen receptor T cells
CD: Cluster of differentiation
CIS: Clinically isolated syndrome
CNS: Central nervous system
CSF-1R: Colony-stimulating factor 1 receptor
Cy: Cyclophosphamide
DC: Dendritic cell
DMT: Disease-modifying therapy
EAE: Experimental Allergic Encephalomyelitis/Experimental Autoimmune Encephalomyelitis
EDSS: Expanded Disability Status Scale
EMA: European Medicines Agency
FMT: Fecal microbiota transplantation
GA: Glatiramer acetate
HERV: Human endogenous retrovirus
HERV-W-ENV: Envelope protein of the human endogenous retrovirus type W
HETA: High-efficacy treatment agent
HSCT: Hematopoietic stem cell transplantation
IFN-β: Interferonβ
IL: Interleukin
iNOS: Inducible nitric oxide synthase
IVIG: Intravenous immunoglobulin
LETA: Low- or moderate-effectiveness treatment agent
MAD: Multiple ascending dose
MBP: Myelin basic protein
MHC: Major histocompatibility complex
MOG: Myelin oligodendrocyte glycoprotein
MRI: Magnetic resonance imaging
MSC: Mesenchymal stem cell
MS: Multiple sclerosis
Nrf2: Nuclear factor erythroid 2-related factor 2
OPC: Oligodendrocyte progenitor cell
PPMS: Primary-progressive multiple sclerosis
RGMa: Repulsive guidance molecule A
rhMOG: Recombinant human myelin oligodendrocyte glycoprotein
RRMS: Relapsing-remitting multiple sclerosis
S1P: Sphingosine-1-phosphate
SAD: Single ascending dose
SPMS: Secondary-progressive multiple sclerosis
TPE: Therapeutic plasma exchange/plasmapheresis
Th: T helper lymphocytes
TNF-α: Tumor necrosis factorα
ZO-1: Zonula occludens 1
